# Central European Early Bronze Age chronology revisited: A Bayesian examination of large-scale radiocarbon dating

**DOI:** 10.1371/journal.pone.0243719

**Published:** 2020-12-28

**Authors:** Mirco Brunner, Jonas von Felten, Martin Hinz, Albert Hafner

**Affiliations:** 1 Institute of Archaeological Sciences, Prehistoric Archaeology, University of Bern, Bern, Switzerland; 2 Oeschger Centre for Climate Change Research (OCCR), University of Bern, Bern, Switzerland; 3 Graduate School Human Development in Landscapes, Kiel University, Kiel, Germany; University at Buffalo - The State University of New York, UNITED STATES

## Abstract

In archaeological research, changes in material culture and the evolution of styles are taken as major indicators for socio-cultural transformation. They form the basis for typo-chronological classification and the establishment of phases and periods. Central European Bronze Age material culture from burials reveals changes during the Bronze Age and represents a perfect case study for analyzing phenomena of cultural change and the adoption of innovation in the societies of prehistoric Europe. Our study focuses on the large-scale change in material culture which took place in the second millennium BC and the emergence at the same period of new burial rites: the shift from inhumation burials in flat graves to complex mounds and simple cremation burials. Paul Reinecke was the first to divide the European Bronze Age (EBA) into two phases, Bz A1 and A2. The shift from the first to the second phase has so far been ascribed to technical advances. Our study adopted an innovative approach to quantifying this phenomenon. Through *regressive reciprocal averaging* and Bayesian analysis of radiocarbon-dated grave contexts located in Switzerland and southern Germany, we modelled chronological changes in the material culture and changes in burial rites in these regions in a probabilistic way. We used kernel density models to summarize radiocarbon dates, with the aim of visualizing cultural changes in the third and second millennium BC. In 2015, Stockhammer et al. cast doubt on the chronological sequence of the Reinecke phases of the EBA on the basis of newly collected radiocarbon dates from southern Germany. Our intervention is a direct response to the results of that study. We fully agree with Stockhammer’s et al. dating of the start of EBA, but propose a markedly different dating of the EBA/MBA transition. Our modelling of radiocarbon data demonstrates a statistically significant typological sequence of phases Bz A1, Bz A2 and Bz B and disproves their postulated chronological overlap. The linking of the archaeological relative-chronological system with absolute dates is of major importance to understanding the temporal dimension of the EBA phases.

## Introduction

In archaeological research, changes in material culture and the evolution of styles are taken as major indicators for socio-cultural transformation. They form the basis for typo-chronological classification and the establishment of phases and periods. As early as 1905, Oscar Montelius assumed in his work "The Method" that in order to clarify not only "chronological" but also "regional" and "social" questions, one must have "a large material basis and a good method" [translation by the author]. Technical innovations were considered a major factor in social transformation [1]. However, the evolutionist idea that past societies adopted innovations immediately is highly problematic and deeply rooted in modern ideas of progress [2]. Paul Reinecke was the first to divide the European Bronze Age EBA into two phases, Bz A1 and A2. Until recently, it has been assumed that A2 followed A1 and represented technical advances [2, 3]. Based on the structure proposed by Reinecke, Ruckdeschel analyzed several graves in Bavaria in 1978. Using the grave goods, especially the pins, he subdivided the two main phases into subcategories (A1a, A1b, A2a, A2b, A2c), which to this day provide the chronological framework of the Early Bronze Age in Central Europe [4].

**Fig 1:**
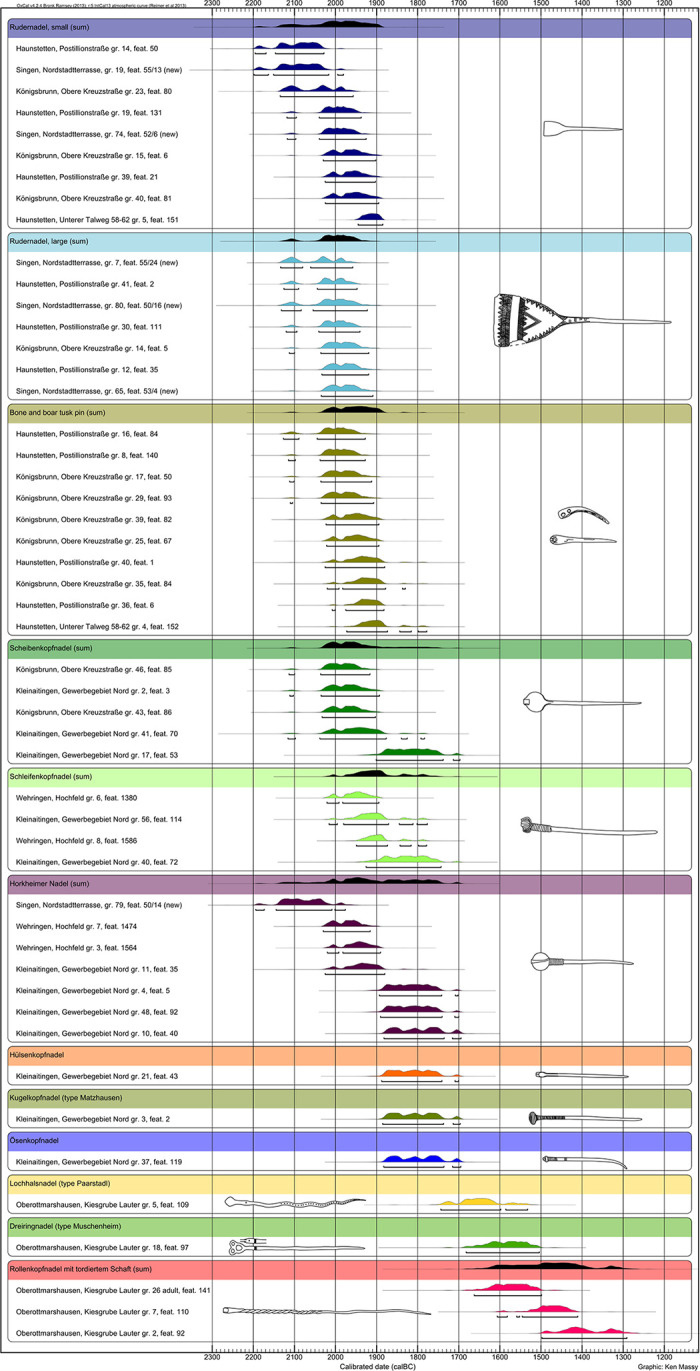
Plot of all pins with radiocarbon dates from Singen and the Augsburg region. Pin types with more than two dates are supplemented with a sum calibration (black) to show their overall timespan [2].

Until the 1980s, the beginning of the Early Bronze Age was dated to 1700 BC by the use of cross dating [2]. In the 1980s, more and more radiocarbon dating was done on graves. In 1988, for the first time, Krause published a larger series of radiocarbon dates from the Singen cemetery in southern Germany [2, 5]. At the same time, dendrochronological data were taken from the wooden chambers of the princely graves at Leubingen and Helmsdorf [2, 6]. In Switzerland, the end of the Early Bronze Age was placed at 1550 BC on the basis of dendrochronological data from lake dwellings [7]. In 2015, for the first time since Krause’s ground-breaking radiometric dating of the EBA graves of Singen, Stockhammer et al. published the results of new large-scale radiocarbon dating of grave finds in the Lech valley near Augsburg in south-eastern Germany [2, 5]. While the primary concern in 1988 was fixing the absolute chronology of the beginning and duration of the EBA stage Bz A1, Stockhammer et al. used radiocarbon data to verify the sequence of phases proposed by Ruckdeschel (1978) [2]. The linking of the relative chronological system with absolute dates is crucial for understanding the temporal dimension of the respective phases. In the opinion of Stockhammer et al., a relative chronological division with the old denominations of Bz A1 and Bz A2 ought no longer to be maintained. On the basis of sum calibrations of radiocarbon dates from graves with pins classifying phases Bz A1b and Bz A2a, the authors assumed that the phases overlapped and that the division of the groups into phases A1 and A2 was therefore not chronological. They concluded that pins of phase Bz A1b could have been in use during the entire EBA and were thus not suitable for defining a Bz A subdivision. They understood the division into groups of forms as a chorological rather than a chronological phenomenon. Stockhammer et al. 2015 referred to the following leading forms for the phases of Ruckdeschel (Fig 1):

- Bz A1a: Rudernadeln (paddle-headed pins with large or small heads), bone pins and boar tusk pins.
- Bz A1a –A1b: Scheibenkopfnadeln (disc-headed pins).
- Bz A1b: Schleifennadeln (knot-headed pins) and Horkheim-type pins.
- Bz A2a: Ösenkopfnadeln (eyelet pins), Hülsenkopfnadeln (pins with sleeve-shaped heads) and Kugelkopfnadeln (globe-headed pins with oblique perforations).

It is worth noting that only the Paarstadl-type pins with perforated shafts (Lochhalsnadel) were removed by Stockhammer et al. from phase Bz A2c and placed at the beginning of the Middle Bronze Age (MBA; Bz B) (Fig 1). While the archaeological material of the Ruckdeschel phases Bz A1a-A2a was well represented in the Augsburg graves, the inventory of phases Bz A2b and Bz A2c was not represented at all. Therefore, no radiocarbon data could be collected for them. Massy et al. 2018 later published three radiocarbon dated burials with Kugelkopfnadeln (globe-headed pins with oblique perforations) of phase Bz A2a-b from Altenmarkt, Osterhofen Am Stadtwald. Graves with roll-headed pins (Rollenkopfnadeln) with twisted necks, three-ring pins (Dreiringnadel) of the Muschenheim type and Paarstadl-type pins with perforated shafts (Lochhalsnadel) were assigned to the MBA Bz B phase. The authors used the 2 σ range of calibration and arrived at the following results with respect to phase chronology:

Bz A1a: 2150/2100–1900 BC
Bz A1b: 2050–1750/1700 BC
Bz A2a: 1900–1700 BC
Bz B: from 1700 BC

Three graves assigned to phase Bz A2a were dated. These contained an eyelet pin (Ösenkopfnadel), a pin with a sleeve-shaped head (Hülsenkopfnadel) and a globe-headed pin with oblique perforation (Kugelkopfnadel). They dated from the period 1900–1700 BC. On the basis of the radiocarbon date from Oberottmarshausen, grave 5, which contained a Paarstadl-type pin with a perforated shaft (Lochhalsnadel), MBA phase Bz B was placed directly after phase Bz A2a. The roll-headed pins (Rollenkopfnadeln) with twisted shafts were also classified as MBA. Accordingly, the authors positioned the change from EBA to MBA at around 1700 BC. Massy 2018 provided a revised version and recalculated the transition around 1650 BC [8].

In order to verify and quantify the statements postulated by Stockhammer et al., which would result, in the original words of the authors, in a ‘Rewriting [of] the Central European Early Bronze Age Chronology’ [2], we applied a multi-method approach. We used radiocarbon dates to detect change/transformation in material culture and burial rites on a macro level [9], focusing on the EBA/MBA periods in southern Germany and Switzerland. Both areas are situated at the northern end of a transect used to investigate transformation processes during the Bronze Age in regions north and south of the Alps. We applied an innovative approach, using Bayesian analysis of radiocarbon-dated grave contexts. Chronological changes in material culture and rituals were modelled in a probabilistic way. We concluded, on the basis of the available dates, that the Bz phases A1 and A2 formed a chronological sequence. Both new radiocarbon dates from the Circum-Alpine EBA graves as well as previous studies from Switzerland and southern Germany contradicted the phaseo-chronological conclusions reached by Stockhammer et al. [10–13] which resulted in their dating of the EBA/MBA transition to around 1700 BC. As a result of our study, which dated the transitions of the phases Bz A1 and Bz A2 by absolute chronology, we placed the EBA/MBA transition at around 1600 BC–a tipping point supported by dendrochronological dates from lake-shore settlements in Switzerland. This chronological correction of the EBA/MBA transition is important for understanding the phaseo-chronological classification of the EBA/MBA and the change in burial rites from inhumation to cremation in the Bronze Age in Europe north of the Alps.

## Materials and methods

In response to Alex Bayliss’s criticism of Bayesian chronological models in archaeology (2015) [14], we consider it essential to summarize both the methodology of the Bayesian methods and data used in this paper [14]. The description of the sample treatment at the Laboratory for the Analysis of Radiocarbon with AMS (LARA) at the University of Bern is attached as S1 File. For the seriation applied to grave goods from graves in southern Germany, an R-code was implemented. In the subsequent methodical treatment, we introduced "regressive reciprocal averaging" seriation. This method provides a more robust seriation, where absolute dating can be included, producing more realistic results than conventional seriation or correspondence analysis.

### Regressive reciprocal averaging

**Fig 2:**
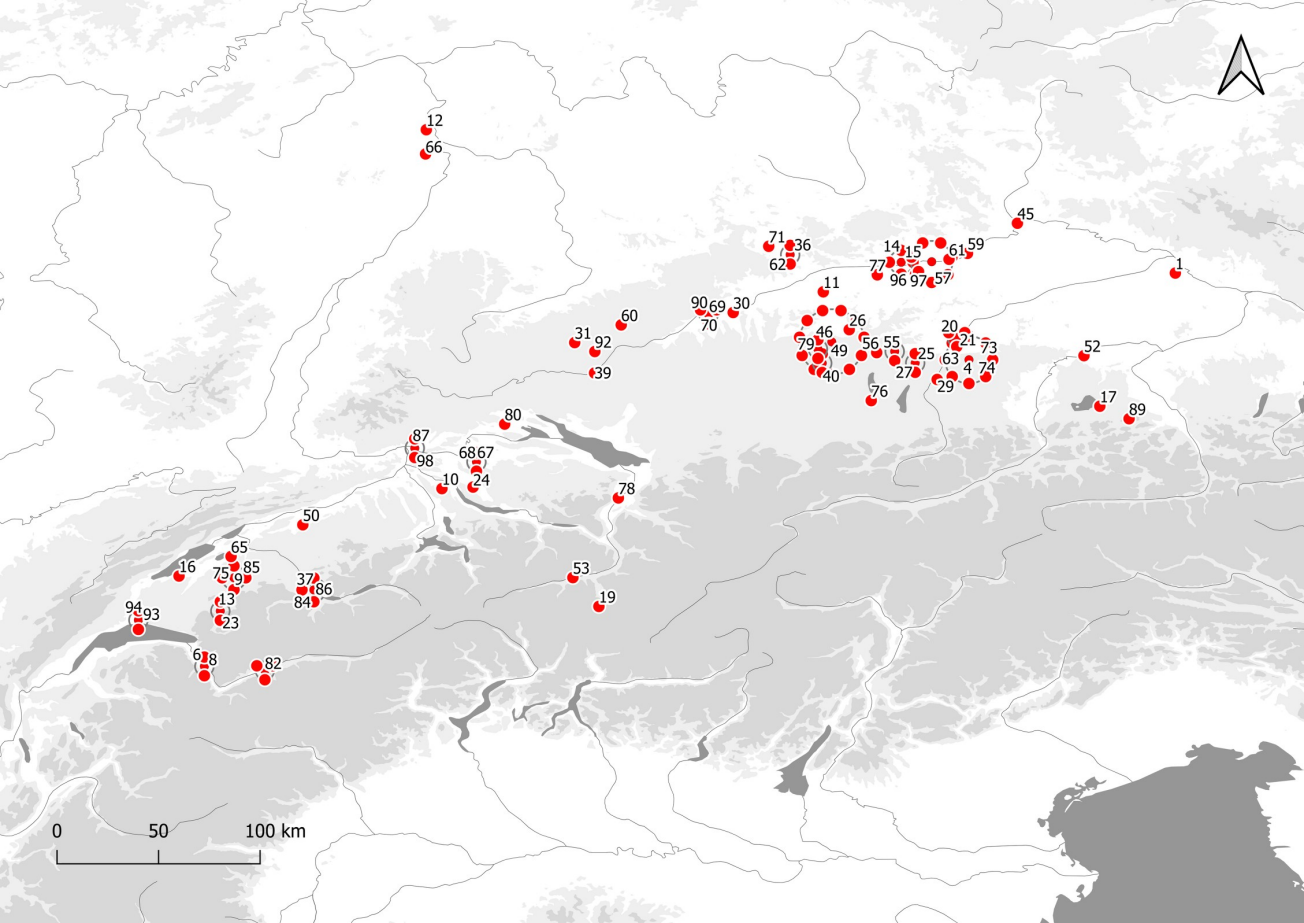
Overview of the sites in southern Germany and Switzerland used for analysis. To provide a better overview, circles are used to represent clusters of dots: 1 Altenmarkt, Osterhofen Am Stadtwald; 2 Aschheim, Feldkirchner Feld; 3 Aschheim, Kita; 4 Aschheim, Sportplatz; 5 Aschheim, Südlich Postfachzentrum; 6 Barmaz I; 7 Bergheim, Förchenaufeld; 8 Bex-les Mûriers; 9 Birch, Düdingen, 10 Birmensdorf, Rameren; 11 Blankburg, Am Burgfeld; 12 Bobenheim-Roxheim, Ernst Roth Straße/Neubau Korz; 13 Bulle, Le Terraillet; 14 Buxheim, Bierwegparallele; 15 Buxheim, Dünzlau; 16 Chables, Les Biolleyres; 17 Chieming, Grabenstätt; 18 Desching, Mühläcker; 19 Donath, Sursés; 20 Eching, BMW-Lager; 21 Eching, Dietersheim; 22 Eching, Hirmerfeld und Liebigstrasse; 23 Enney, Le Bugnon; 24 Fällanden, Fröschbach; 25 Freiham, Gut Freiham; 26 Friedberg, Metzgerwäldchen; 27 Germering, Breslauer Strasse; 28 Grossmehring, Strassgwender; 29 Grünwald, Gymnasium; 30 Günzburg, Ulmer Strasse; 31 Harthausen b. Feldhausen, Bühl, Hügel 1; 32 Haunstetten, Postillionstrasse; 33 Haunstetten, Unterer Talweg 109-111-113; 34 Haunstetten, Unterer Talweg 58–62; 35 Haunstetten, Unterer Talweg 85; 36 Heroldingen, Hoppingen; 37 Hilterfingen, Schlosspark Hünegg; 38 Hilterfingen, Im Äbnit/Tannbühlstrasse; 39 Hundersingen, Weidenhang, Hügel; 40 Hurlach, Mitterfeld; 41 Ingolstadt-Mailing, MIBA-Gelände; 42 Ingolstadt, Carraraplatz; 43 Kleinaitingen, Gewerbegebiet Nord; 44 Kleinaitingen, Herbst- und Friedenstrasse; 45 Kleinprüfening, Buchschlag; 46 Königsbrunn, Afra- und Augustusstrasse; 47 Königsbrunn, Kiesgrube Burkhart; 48 Königsbrunn, Obere Kreuzstrasse; 49 Königsbrunn, Simpertstrasse; 50 Koppigen, Usserfeld; 51 Kösching, Frühlingsstrasse; 52 Kraiburg, Römerstrasse; 53 Laax, Salums; 54 Maisach-Gernlinden, Südumgehung; 55 Maisach, Frauenstrasse; 56 Mammendorf, Bürgermeister-Drexler-Bogen; 57 Manching, Hundsruckenacker; 58 Manching, Westenhausen; 59 Marching, Gangsteig; 60 Mehrstetten, Oberes Häule Hügel 2; 61 Menning, Bachberg; 62 Möttingen, Baadfeld; 63 München-Trudering, Friesen- und Karpfenstrasse, 64 München, Stegmühlstrasse; 65 Murten, Löwenberg; 66 Mutterstadt, Auf dem Limburg; 67 Neftenbach I (Steinmöri); 68 Neftenbach II (Zürichstrasse 55); 69 Nersingen, Leibi, Steinegert; 70 Nersingen, Leibi; 71 Nördlingen-Baldingen, am Mühlweg Ost; 72 Oberottmarshausen, Kiesgrube Lauter; 73 Poing, Nord; 74 Poing, Siemensgelände; 75 Posieux, Châtillon; 76 Raisting, Langpommer-Äcker; 77 Rohrenfels; 78 Rüthi, Hirschensprung; 79 Schwabmünchen, Mittelstetten; 80 Singen am Hohentwiel; 81 Sion, Petit-Chasseur I; 82 Sion, Petit-Chasseur II; 83 Sion, Petit-Chasseur III; 84 Spiez-Einigen, Holleeweg 3; 85 Tafers, Kiesgrube Zelgli; 86 Thun, Wiler; 87 Tiengen, Eidöre/Auf dem Buck, Hügel A; 88 Triesen, Fürst-Johann-Strasse 40; 89 Unterbrunnham, Wagenau Hügel 16; 90 Unterelchingen, Obstgartenstrasse; 91 Untermeitingen; 92 Upflamör, Lautrieb Hügel 11; 93 Lausanne, Vidy; 94 Vufflens-la Ville, En Sency; 95 Wehringen, Hochfeld; 96 Weichering, Toter Mann; 97 Zuchering, Süd; 98 Zurzach, Schlosspark Himmelreich. Basic vector map of Europe; the isohypses were produced by using Copernicus data and information funded by the European Union—EU-DEM layers https://doi.org/10.5281/zenodo.3457998 [17].

For the analysis of 135 radiocarbon-dated EBA graves from southern Germany we used the data from Stockhammer et al. 2015 [2], Massy 2018 [8], Massy et al. 2018 [15] and Massy and Stockhammer 2019 [16]. 123 graves were from eight cemeteries of the Lech Group, 10 were from the cemetery at Singen, two from different cemeteries of the Ingolstadt Group and three from Altenmarkt, Osterhofen Am Stadtwald (Fig 2).

Each EBA grave was recorded as a single assemblage. We are of the opinion that single objects are not representative of phases and therefore all finds from the grave were considered as a single complex [18]. In the case of individual burials and cremation burials, we assumed that all the associated grave goods were placed in the grave at the same time. Particular importance was attached to metal, bone and antler jewelry and selected objects such as silex arrowheads or flanged bronze axes, since these grave goods were identified by Ruckdeschel as chronologically relevant and were frequent enough to allow seriation [4]. In addition, since graves with burials in a stretched supine position usually had no or only a few grave goods, the position of the dead was taken into account. To analyze the data, we used a multivariate statistical method, weighted according to the available radiocarbon data. A common method for creating a radiocarbon data-weighted seriation is Canonical Correspondence Analysis [19]. With this method, however, the weighting cannot be adjusted. Moreover, only single events can be used for weighting, not the time spans specified by radiocarbon dates. Regressive reciprocal averaging, a modified version of reciprocal averaging, was therefore used instead (S2 File). This method has two advantages: firstly, calibrated radiocarbon data can be used for seriation weighting; secondly, the weighting can be customized.

In the case of a table of assemblage items, unmodified reciprocal averaging involves swapping and normalizing the rows and columns according to their column or row index, and multiplying by the weighted average of the column/row, which results in the reordering of the table. These steps are repeated until almost no changes occur and a diagonalized table is created [20].

To weight reciprocal averaging according to available radiocarbon data, regressive reciprocal averaging requires, in addition to the table of find complexes, an auxiliary table that represents the relevant calibrated radiocarbon data. The second table has a fixed order of columns, representing time periods, so that only the rows must be reordered. In each iteration step, the new row indices are calculated for both tables, in addition to the column indices for the table of find complexes. The columns of the table of find complexes are reordered without taking the auxiliary table into account, but the row indices are averaged between the two tables using a factor to weight the influence of the radiocarbon table. These steps are repeated until almost no changes occur, as in the case of unmodified reciprocal averaging. The results are two tables, the diagonalized table of find complexes and a table with the corresponding radiocarbon data. The radiocarbon table can be used to check visually whether a chronological sequence of graves is possible or not, and, if necessary, to adjust the weighting of the data. The resulting chronological order of the graves can be validated through Bayesian modelling after typological phases have been established.

After regressive reciprocal averaging, find complexes were divided into phases. Again, only typologically assignable find complexes were considered. In addition, typologically relevant radiocarbon-dated graves of the Central Alpine region and MBA graves of southern Germany were integrated into the corresponding phases (S1 Data). For the analysis of the radiocarbon dates, we selected only those which were reliable and clearly associated with the archaeological event being dated. All the data used in this paper were taken from the radiocarbon database Radon-b and are summarized in S1 Data [21].

### Radiocarbon dating

**Fig 3:**
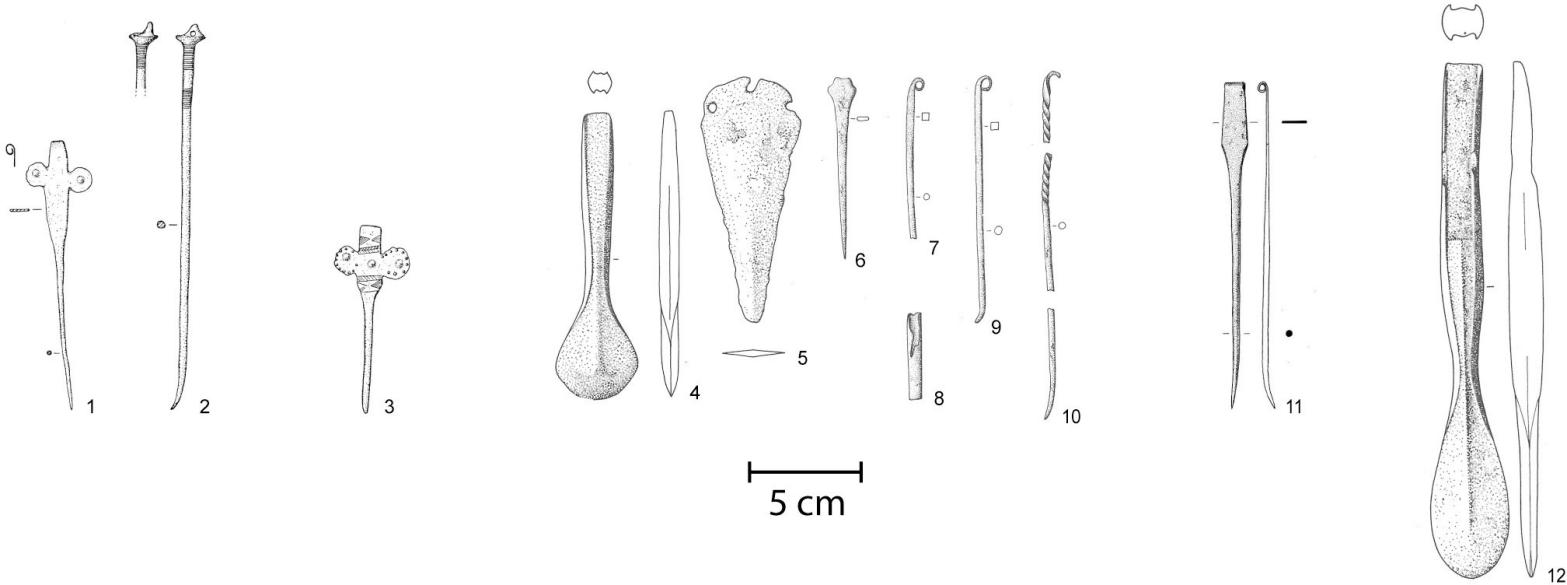
Newly radiocarbon-dated graves. 1–2 Donath, Sursés grave 3A; 3 Donath, Sursés grave 3B; 4–10 Enney, Le Bugnon grave 1; 11 Posieux, Châtillion; 12 Tafers, Kiesgrube Zelgli. Drawings 1–3 after Spindler 1973 [27]; 4–10 after Abels 1972 [28]; 11 after Ramseyer 1990 [29]; 12 after Abels 1972 [28]. Scale 1:4.

Additionally, the graves of Donath-Sursés, Laax-Salums, Tafers-Kiesgrube Zelgli, Enney-Le Bugnon and Posieux-Bois de Châtillon were radiocarbon dated at the Laboratory for the Analysis of Radiocarbon with AMS (LARA) at the University of Bern (Table 1) [22]. The sample pretreatment of human bones was based on protocols described in Szidat et al. 2017 [23]. The description of the sample treatment at the Laboratory for the Analysis of Radiocarbon with AMS (LARA) at the University of Bern is attached as S1 File. The samples for the radiocarbon dating were loaned from various institutions and are archived and protected by them. The samples of the graves from Donath, Sursés (Donath_361_A, Donath_361_B, Donath_362_A, Donath_362_B, Donath_363_A, Donath_363_B, Donath_1922_A, Donath_1922_B, Donath_1923_A, Donath_1923_B) were loaned by the Interkantonale Arbeitsgemeinschaft zur Betreuung anthropologischer Funde (IAG), University of Basel, Institut für Integrative prähistorische und naturwissenschaftliche Archäologie (IPNA). The samples of Laax-Salums (LS_1979_Zone EII_A, LS_1979_Zone E_II_B) come from the archive of the Archaeological Service of Grisons, Switzerland. The samples of the graves Enney, Le Bugnon (EN-BU 1942), Posieux, Châtillon (PO-CHA/FE) und Tafers, Kiesgrube Zelgli (TA-ZE 1935) are from the archive of the Service Archéologique de l'Etat de Fribourg (SAEF), Switzerland. The sample labels are correlated with the lab code on Table 1. In this article, we present 15 new radiocarbon dating results for EBA inhumations (Fig 3 and Table 1). The classification of the grave goods into the corresponding phase was made after David–Elbiali 2000 [24]. It should be noted that the phase BzA2c as defined by Reinecke has not received acceptance in Swiss research and is generally referred to as BzA2b [24–26]. BzA2 is therefore the last Early Bronze Age phase before the Middle Bronze Age phase BzB.

**Table 1:** Results of new radiocarbon dating of Early Bronze Age graves in Switzerland.

Site, grave	Sample label	Lab code	Material	14C age (BP)	±	C %	C:N	Gelatin yield (% w/w)	Reinecke Phase
**Donath, Sursés Grave 2A**	Donath_361_A	BE-6714.1.1	bone	3326	34	11.2	3.24	5.9	indet.
**Donath, Sursés Grave 2A**	Donath_361_B	BE-6715.1.1	bone	3290	20	17.3	3.3	7.9	indet.
**Donath, Sursés Grave 2B**	Donath_362_A	BE-6712.1.1	bone	3323	45	6.9	3.27	8.6	indet.
**Donath, Sursés Grave 2B**	Donath_362_B	BE-6713.1.1	bone	3270	37	8	3.23	5.9	indet.
**Donath, Sursés Grave 2E**	Donath_363_A	BE-6716.1.1	bone	3437	21	9.2	3.25	7.6	indet.
**Donath, Sursés Grave 2E**	Donath_363_B	BE-6717.1.1	bone	3371	42	3	3.32	14.6	indet.
**Donath, Sursés Grave 3A**	Donath_1922_A	BE-6718.1.1	bone	3289	36	4.5	3.36	11.6	Bz A2b
**Donath, Sursés Grave 3A**	Donath_1922_B	BE-6719.1.1	bone	3358	36	4.9	3.25	11.3	Bz A2b
**Donath, Sursés Grave 3B**	Donath_1923_A	BE-6722.1.1	bone	3294	36	4.9	3.25	10.1	Bz A2a
**Donath, Sursés Grave 3B**	Donath_1923_B	BE-6723.1.1	bone	3352	35	5.1	3.32	8.9	Bz A2a
**Enney, Le Bugnon**	EN-BU 1942	BE-6982.1.1	bone	3474	19	11.5	3.5	2.6	Bz A2a
**Laax-Salums 1979_Zone E II_A**	LS_1979_Zone EII_A	BE-6913.1.1	bone	3242	19	4.4	3.46	9.33	indet.
**Laax-Salums 1979_Zone E II_B**	LS_1979_Zone E_II_B	BE-6914.1.1	bone	3180	33	4.2	3.54	8.9	indet.
**Posieux, Châtillon**	PO-CHA/FE	BE-6983.1.1	bone	3528	18	23.1	3.3	3	Bz A2a
**Tafers, Kiesgrube Zelgli**	TA-ZE 1935	BE-6981.1.1	bone	3424	18	12.2	3.3	15	Bz A2b

Dated at the LARA Laboratory of the University of Bern.

### Bayesian modeling of large-scale radiocarbon dating

In archaeology, absolute dating is an indispensable basis for understanding the evolution and dynamics of cultural phenomena. Only by dating independently from archaeological typology it is possible to understand typological development itself [30]. For evaluations exceeding the intra-site level, it is particularly important that data is collected in large numbers and that the dates are easily accessible. Modern statistical analyses, such as sequential calibration based on Bayesian methods, also require, not single dates, but large numbers of dates [31]. By combining large amounts of data, far more sophisticated results can be achieved than by using conventional evaluations [2, 8] (e.g. [32]). Usually, radiocarbon ages are converted into calendar ages that are given as confidence intervals [33]. It may happen that more than one radiocarbon age corresponds to a specific calendar date, resulting in rather larger overall confidence intervals. Attempts to reduce these intervals must be approached in a quantitative way. Bayesian analysis is a useful tool for dealing with such problems and can determine confidence intervals and probability distributions for the calibrated radiocarbon dates. Information about chronology can be transformed into explicit statistical estimates for the dates of past events [34].

In a frequentist approach, the event of interest is a possible outcome of a random experiment that can be reproduced infinitely, each experiment being capable of producing independent results. The observed data are repeatable random samples and by using specific inference methods, they can be suitably fitted to a theoretic probability distribution, which may depend on one or more parameters. In classical statistics, these parameters remain constant during the repeatable process and, if they are unknown, their real values can also be estimated using either point estimates or confidence intervals. With Bayesian analysis, on the other hand, the parameters are no longer assumed to be constants, but rather random variables, having their own probabilistic distribution. The Bayesian approach is to determine the posterior distributions of the unknown parameters, given available data or some prior information about these parameters. The determination of the posterior conditional distribution of the parameter is based on the information available and on the user’s decision as to the best estimate of the parameter at that time. In archaeological sciences, the parameters are usually calendar dates of events and the data consist of certain fixed observations selected for estimating the parameters [35]. In radiocarbon dating chronology, there are two types of applicable model (for more details, see [14, 36, 37]):

- A stratigraphic order model, used when there are strong reasons for considering a chronological order to a series of events. This prior information may originate from historical records or from scientific evidence like stratigraphies. Once a chronological order is set, this should strongly affect the outcome of the calendar dates obtained.
- If no historical or stratigraphic information is available, the prior information is based on assumptions about the probability distribution of dates in a single phase of activity. Typically, the prior distribution should include all plausible values for the unknown parameter.

Adopted by researchers for use in archaeological applications over two decades ago (e.g., [34, 38–40, 41–43] Bayes’ theorem can be expressed mathematically as follows:

p(t|y)αp(t|y)p(t)

where *t* represents a set of parameters, *y* represents observations or measurements, *p*(*y|t*) is the likelihood, and *p*(*t|y*) is the posterior probability, or the probability of a given parameter set given the measurements and the priors [37]. This is expressed in a simpler manner by Bayliss 2007 [44] and reads as follows:

$$\frac{P(data|parameters)}{P(data)}\times P(parameters)=P(posterior|data)$$

where the likelihood is determined by the probability of the data or observations given the set parameters and is proportional to the probability of the parameters themselves. The combination of these two observations/measurements and prior information or beliefs is where the value of Bayesian statistical methods lies, especially in regard to interpreting radiocarbon data [45].

In order to determine the functional shape of the likelihood function, it is usual to assume that the observations belong to a prescribed interval (timescale). By specifying how likely the dates within the interval are, we obtain a probability distribution function for the data. In OxCal 4.4 one can use different boundaries to define the type of probability distribution used [37]. In order to streamline our approach, we used the model of multiple phases in a sequence, within which we applied the contiguous phase model, which assumes a linear continuity of data or groups of data. Transition boundaries were positioned between the phases. This hypothesis does not preclude a parallel existence for a period.

For our analysis, each posterior distribution was given an agreement index (A) which is displayed on the plot with the sampled distribution name. A indicates the extent to which the final (posterior) distribution overlaps with the original distribution (S2 Data). An unaltered distribution has an A index of 100% but it is possible for the value to rise above this if the final distribution only overlaps with the very highest part of the prior distribution. If the A value is below 60% for any individual item, it may be worth questioning its position in the period and an error message is generated (this level of disagreement is very similar to that for the 5% level chi squared test). For a group of items (such as a sequence) it is possible to define an overall agreement index which is a function of all of the indices within the group. If this falls below 60% it may be worth re-evaluating the assumptions made. This overall agreement is shown on the plot at the top of the sampled group and will be in a form like: Sequence {A = 100.9%(A'c = 60.0%)}where A is the calculated overall agreement index and A'c is the level below which it is not expected to fall [37].

**Fig 4:**
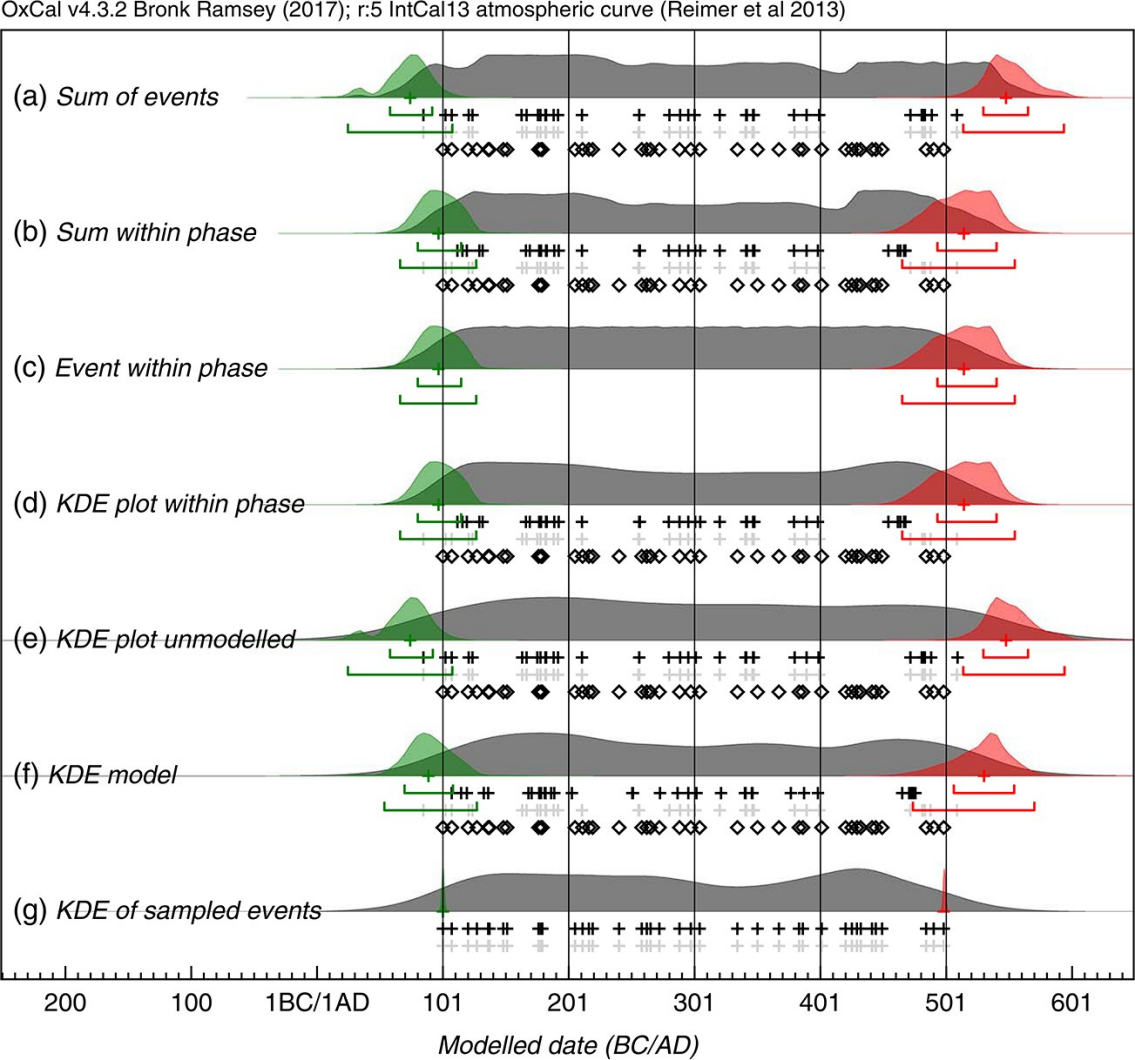
Comparison of methods for summarizing a set of 40 radiocarbon dates. The open diamonds show the randomly selected dates in the range AD 100–AD 500, the light gray crosses show the medians of the likelihood distributions of the calibrated dates and the black crosses the medians of the marginal posterior distributions for each dated event. Panel (a) shows the sum of the likelihoods. Panels (b), (c), and (d) all use the marginal posteriors from the same simple single uniform phase model with a start and end boundary (Bronk Ramsey 2009 [37]): panel (b) shows the sum of the marginal posteriors, panel (c) shows the marginal posterior for an event simply constrained to lie between the start and end boundary, and panel (d) shows a kernel density plot based on the dated events constrained to be within the phase. Panel (e) is a KDE plot generated from samples randomly taken from the likelihood distributions. Panel (f) shows the effect of applying the KDE_Model model which uses the KDE distribution as a factor in the likelihood (see text). Panel (g) shows a kernel density plot of the original calendar dates chosen from the range AD 100–AD 500: ideally this is the distribution that the other estimates should reproduce. The overlaid green and red distributions with their associated ranges show the marginal posterior for the First and Last events within the series: these should overlap at 95% with the first and last open diamonds which are the actual first and last events sampled; this is the case for those based on the uniform phase model and the KDE model but not those based on the unconstrained Sum or KDE plot. Graphic credits Bronk Ramsey, 2017 [46].

As a last step we used KDE_Plots within each phase for summarizing radiocarbon dates, instead of Sum Distributions as suggested by Bronk-Ramsey 2017 [46]. If the processes underlying the data are properly understood, it is possible to overcome the excessive spread problem by employing a Bayesian model. The simplest method is to use a single uniform phase model. It is then possible to calculate the sum or the kernel density of the marginal posterior distributions of the events within the phase. This provides a visualization of the overall distribution of dated events within the phase [46]. The advantages and disadvantages of this method are discussed in Bronk-Ramsey 2017 and illustrated with examples (Fig 4) [46].

It is useful to consider what the summed distribution actually represents for a set of samples. If a single sample is selected at random and the probability density for the age of that sample is required, then the normalized summed distribution is appropriate. If the likelihood distributions from the calibration are summed, then the assumption is that all of the measurements are independent and that there is no a priori reason to suppose the events are related in any way [46].

Using the KDE_Plot method provides a display of the distribution of events which is free from the noise artifacts seen in Sum plots (Fig 4). However, unless the plotting method is used together with another Bayesian model (Fig 4), the distributions are over-dispersed (Fig 4D) and smear the underlying signal (Fig 4). This has nothing to do with the method itself; it arises from the assumption that each parameter is independent, which makes a greater spread statistically much more likely. This overall approach for the KDE model is an extension to that used for the KDE_Plot, where the KDE was used only to estimate the distribution of undated events. From a Bayesian perspective, the prior for each dated event is the KDE distribution for all of the other events, and the prior for all undated events is the KDE for all of the dated events. Because the KDE method is in itself frequentist, this is not a purely Bayesian approach. Other Bayesian methods, such as the Bayesian Bootstrap [47], could be used, but all require information on the distribution characteristics. If it is accepted that the KDE is a generally reasonable estimate for any underlying density when we have randomly selected samples, then the approach taken here is a reasonable approach for dealing with densities of events for which we have little or no quantitative prior knowledge [46].

Because different burial rites cannot necessarily be assigned to certain periods of time but may, rather, occur very heterogeneously in space and time, a KDE_model seems a suitable method for determining patterns. The KDE_Model algorithm in OxCal can be tested against the same simulated uniformly distributed data as used with the Sum method, as shown in Fig 4. The output of this model, together with the sampled distributions for the First and Last events, is shown in Fig 4, where it can be seen that the overall span is much closer to the original, and to the output of the single uniform phase model (Fig 4B–4D), than either the Sum plot (Fig 4A) or the KDE_Plot (Fig 4E). The algorithm removes high frequency noise in the form of sharp edges, peaks and troughs but retains the lower frequency signal [48]. Compared to the uniform phase model, which specifically assumes abrupt boundaries, the method is limited in detecting abrupt ends to the true distribution, as is revealed by the more sloping tails of the distribution and the marginally wider estimates for the first and last event. However, for the randomly selected calendar events, the method does provide an output similar to that of the KDE_Plot (Fig 4G), which is the objective of the method. The KDE_Model was implemented in OxCal. For the kernel and factor we used the default to N(0,1) and U(0,1), as for the KDE_Plot function above [46]. This method is explained in detail in Bronk-Ramsey 2017 [46].

## Results

**Fig 5:**
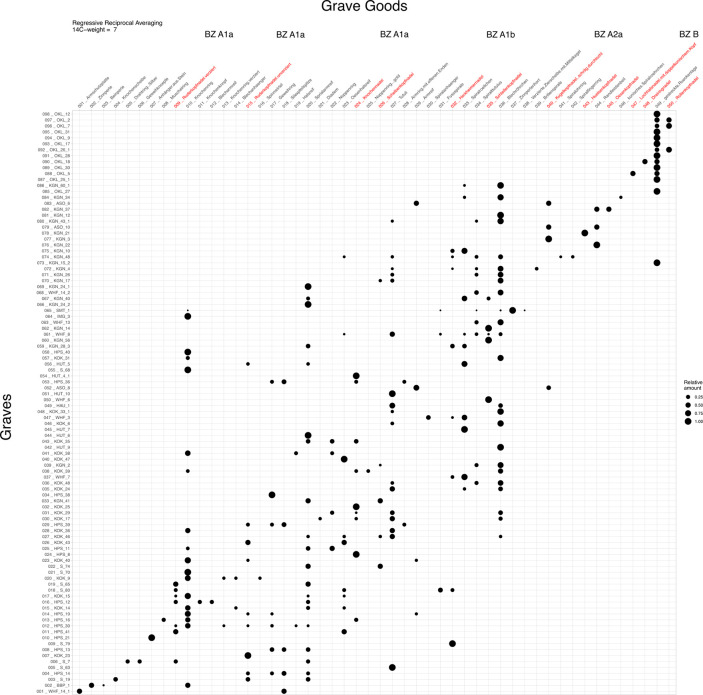
Results of the regressive reciprocal averaging seriation of the radiocarbon-dated burials from southern Germany. Y axis: graves; X axis: finds; point size: percentage of finds in the grave.

In this paper we present the results of the radiocarbon dating in chronological order for the Central Alpine region and southern Germany from the EBA to the MBA. We combine them with the traditional archaeological division of the respective phases in order to connect traditional relative chronological phases with the absolute-chronological evidence. Since the EBA graves of southern Bavaria allow a seriation of the individual burials, the graves were analyzed in a multivariate statistical procedure. Using a reciprocal averaging method, the individual graves were seriated and weighted according to their radiocarbon dating (S2 File). The results of the analysis show a sequence of graves comparable to the seriation of Ruckdeschel 1978 [4]. His sequencing of the individual phases on the basis of the grave goods is confirmed by our regressive reciprocal averaging seriation (Fig 5). Therefore, we assume that the separation of the Bz phases A1 and A2 is chronological.

According to the results of the regressive reciprocal averaging seriation we assume that certain types of grave goods were deposited in the burials over certain periods of time. To verify this hypothesis, we used 256 radiocarbon-dated graves from the EBA and MBA originating from 98 archaeological contexts (S1 Data). The radiocarbon dates from Southern Germany all originate from human bones. For the dating from Switzerland, dating of human bones as well as charcoal was considered. For the Bronze Age graves from Switzerland, charcoal samples had to be used in some cases, as the preservation of the bones was not always guaranteed. It should be noted that charcoal always bears the risk of old wood effect. The selection of charcoal dates was limited to reliable samples as already proposed for the Swiss data in Capuzzo and Barceló 2015 [49].

**Fig 6:**
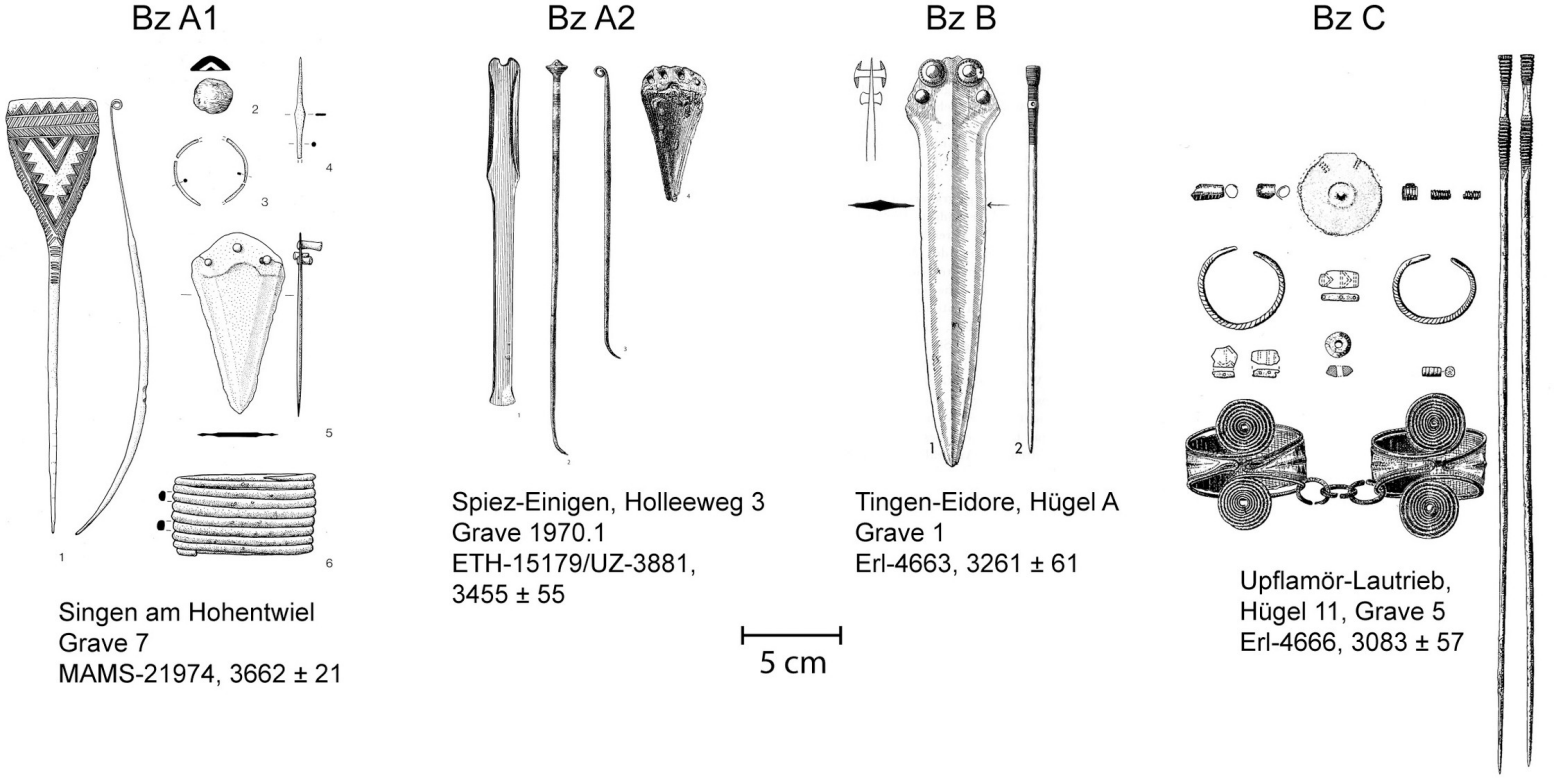
A selection of graves from the Early and Middle Bronze Age phases. Drawings after Krause 1988 [5], Hafner and Suter 1998 [10], Kimmig and Unser 1954 [50] and Müller and Lorke 2009 [13]. Scale 1:4.

The graves had been classified and assigned to phases by Ruckdeschel 1978 [4], Krause 1988 [5], David-Elbiali 2000 [24], Müller and Lorke 2009 [13], Stockhammer et al. [2] Massy [8] and Massy et. al. [15]. The following criteria were applied for the classification of the phases (Fig 6):

- Bz A1: paddle-headed pins (Ruderkopfnadel), perforated bone pins (durchlochte Knochennadel), disc-headed pins (Scheibenkopfnadel), knot-headed pins (Schleifenkopfnadel) and Horkheim-type pins
- Bz A2: eyelet pins (Ösenkopfnadel), pins with sleeve shaped heads (Hülsenkopfnadel), pins with cloverleaf-shaped heads (Flügelnadel) with or without decoration, pins with rhomb-shaped heads (Rautennadel) and globe-headed pins with oblique perforation (Kugelkopfnadel)
- Bz B: pin types with perforated shafts (Lochhalsnadel).
- Bz C: pins with richly decorated proximal ends, ribbed pins with slightly flared heads and richly decorated proximal ends, seal-headed pins (Petschaftskopfnadel).

First, we analyzed dated graves in order to detect chronological differences. For our observations we used 125 radiocarbon dates from our dataset (S1 Data and Fig 2), selected from graves with pins that could be assigned to one BA phase. In order to detect the time span of the transitions between the relative chronological phases of the EBA and MBA, the samples were ordered in four groups according to their typological phases, based on their grave goods (S1 Data). In each phase group, the samples were distributed in chronological order, from oldest to youngest. We ran a phase model (contiguous) with OxCal v 4.4 [37, 51]. The application calculated Gaussian transition boundaries between the phases and provided this information according to 1σ and 2σ probabilities. To calculate the time span of each phase, we first used a sum calibration within each phase and then compared this to the kernel density plot within each phase as explained above. Another important aspect of the model was the identification of the outliers. A date can be defined as an outlier when the agreement index A is less than 60 per cent [52]. In such cases, the confidence interval of the date does not statistically fit into the phase it was assigned to.

**Fig 7:**
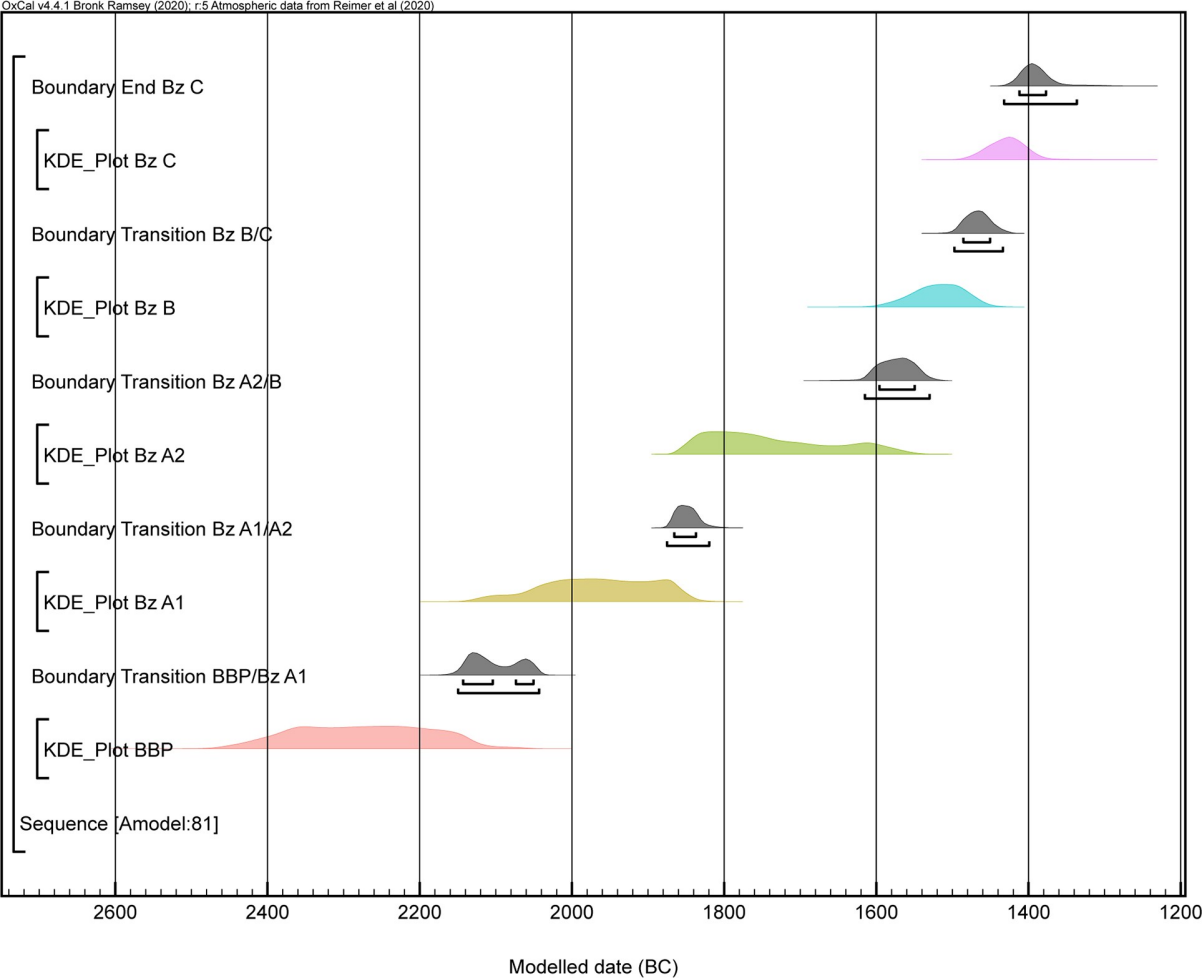
Highest posterior density intervals for the estimated start and end of each Bronze Age phase and KDE plot visualization of the overall distribution of dated events within each phase. These are derived from a Bayesian model defined by the OxCal code provided as supplementary information. The individual dates are constrained by the information incorporated in the model.

Of the data set of 125 dates we modelled, 33 were from the Central Alpine area and 94 from southern Germany (Fig 7 and S3 File). The dates were divided into five phases: Bell Beaker Phenomenon, Bz A1, Bz A2, Bz B and Bz C (Table 2).

**Table 2:** Modelled time spans for each phase and transition after S2 Data.

Period	Modelled time span (BC, with 95.4% probability)
Bz C	1483–1364
Transition Bz B/C	1498–1434
Bz B	1595–1444
Transition A2/B	1616–1531
Bz A2	1865–1546
Transition Bz A1/A2	1876–1820
Bz A1	2134–1834
Transition Bz BBP/ Bz A1	2150–2044
BBP	2470–2061

**Fig 8:**
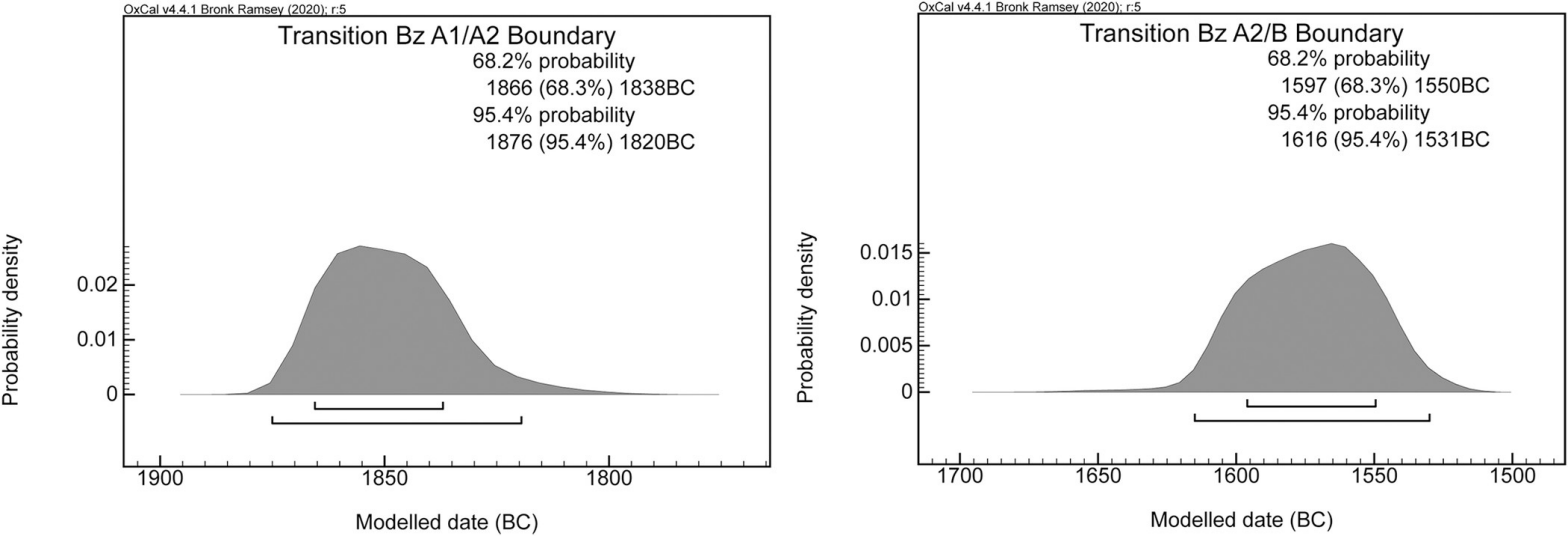
Gaussian boundary of the transitions between Bz A1 and A2 and Bz A2 and B; both 1σ and 2σ probability intervals are marked.

The proximity of the A_model_ (81.2) and A_overall_ (78.9) indexes was very good and suggested a strong consistency for the proposed model. The data were distributed as follows: The BBP and Bz A1 phases included mostly the data of Stockhammer et al. 2015 and Massy 2018 (S1 Data) [2]. The modelled dates were situated between 2470 and 2060 BC. The transition from the BBP to Bz A1 lay around 2150–2045 BC. This result is comparable to the supposed transition between the Bell Beaker Phenomenon and the EBA [2]. A time span of 2135–1835 BC could be calculated for phase Bz A1. The transition from phase BzA1 to phase BzA2 was 1875–1820 BC (Fig 8). The model showed that the two stages could be separated from each other. Phase Bz A2 had a range of 1865–1545 BC. The transition from the EBA to the MBA could therefore be set at 1615–1530 BC (S2 Data and Fig 8).

The MBA phases Bz B and Bz C could also be separated on the basis of the radiocarbon dates. Phase Bz B had a time span of 1595–1445 BC. The transition from Bz B to Bz C, according to our model, was 1500–1435 BC (Table 2 and S2 Data). The phase Bz C dated from 1485 to 1365 BC.

The model had a total of three outliers. The first was from Hilterfingen-Im Aebnit Tannenbühlstrasse (ETH-15183/UZ-3885, 3600 ± 50) where the dating was seen as suspicious and too old from the first attempt [10] (S2 Data). The other two were from Obermattshausen, graves 2 (MAMS-21546, 3132 ± 42) and Altenmarkt, Osterhofen Am Stadtwald Grab 8 (MAMS-30971, 3583±23).

**Fig 9:**
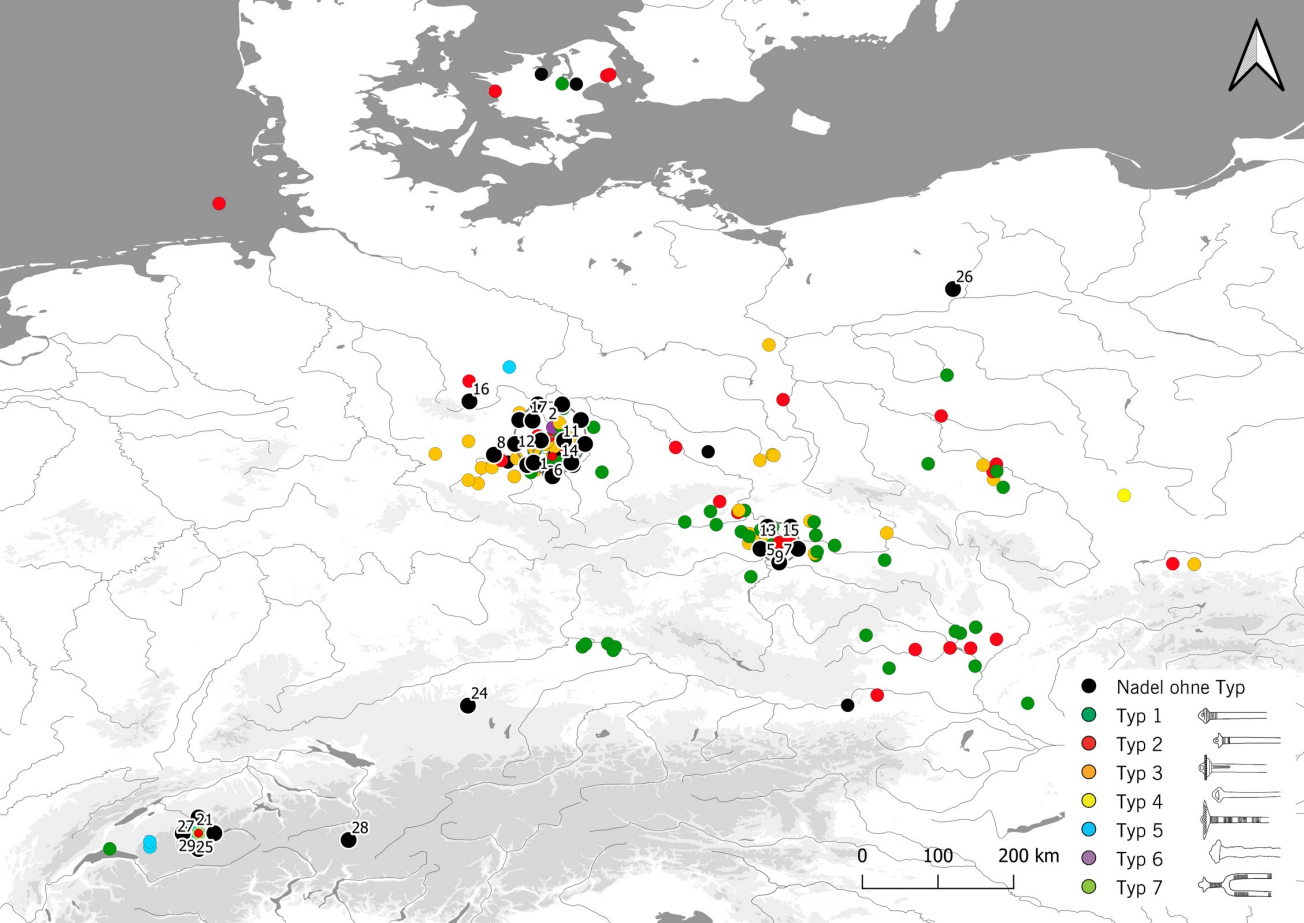
Distribution of Bronze Age eyelet pins from graves in Europe. 1 Eulau, cemetery 3, grave 4 (Bef. 338); 2 Schiepzig; 3 Eulau, cemetery 7, Bef. 1; 4 Oechlitz, grave Bef. 25942; 5 Praha-Miškovice, grave 8; 6 Eulau, cemetery 4, grave Bef. 492; 7 Praha-Miškovice, grave 21; 8 Leubingen, central grave; 9 Praha-Miškovice, grave 19; 10 Stöbnitztal/Oberwünsch, ICE-Trasse, grave (rechter Hocker), Bef. 290; 11 Dörstewitz/Schkopau, grave Bef. 80189; 12 Stedten; 13 Praha-Miškovice, grave 33; 14 Dörstewitz/Schkopau, grave Bef. 80198; 15 Praha-Miškovice, grave 32; 16 Benzingerode, grave 14; 17 Helmsdorf, central grave; 18 Eulau, cemetery 3, grave 5 (Bef. 342); 19 Eulau, cemetery 3, grave 5 (Bef. 342); 20 Bad Lauchstädt, grave Bef. 60483; 21 Spiez-Einigen, grave 2; 22 Bad Lauchstädt, grave Bef. 60481; 23 Bad Lauchstädt, grave Bef. 60651; 24 Kleinaitingen, Gewerbegbiet Nord grave 37; 25 Spiez-Einigen, grave 1; 26 Krajeńskie, Hexenberg, grave 91; 27 Spiez-Einigen, grave 2008.1; 28 Donath-Surses, grave 3A; 29 Spiez-Einigen, grave 2008.2. The data are from Knoll and Meller 2015 [48]and Massy 2018 [8].

**Fig 10:**
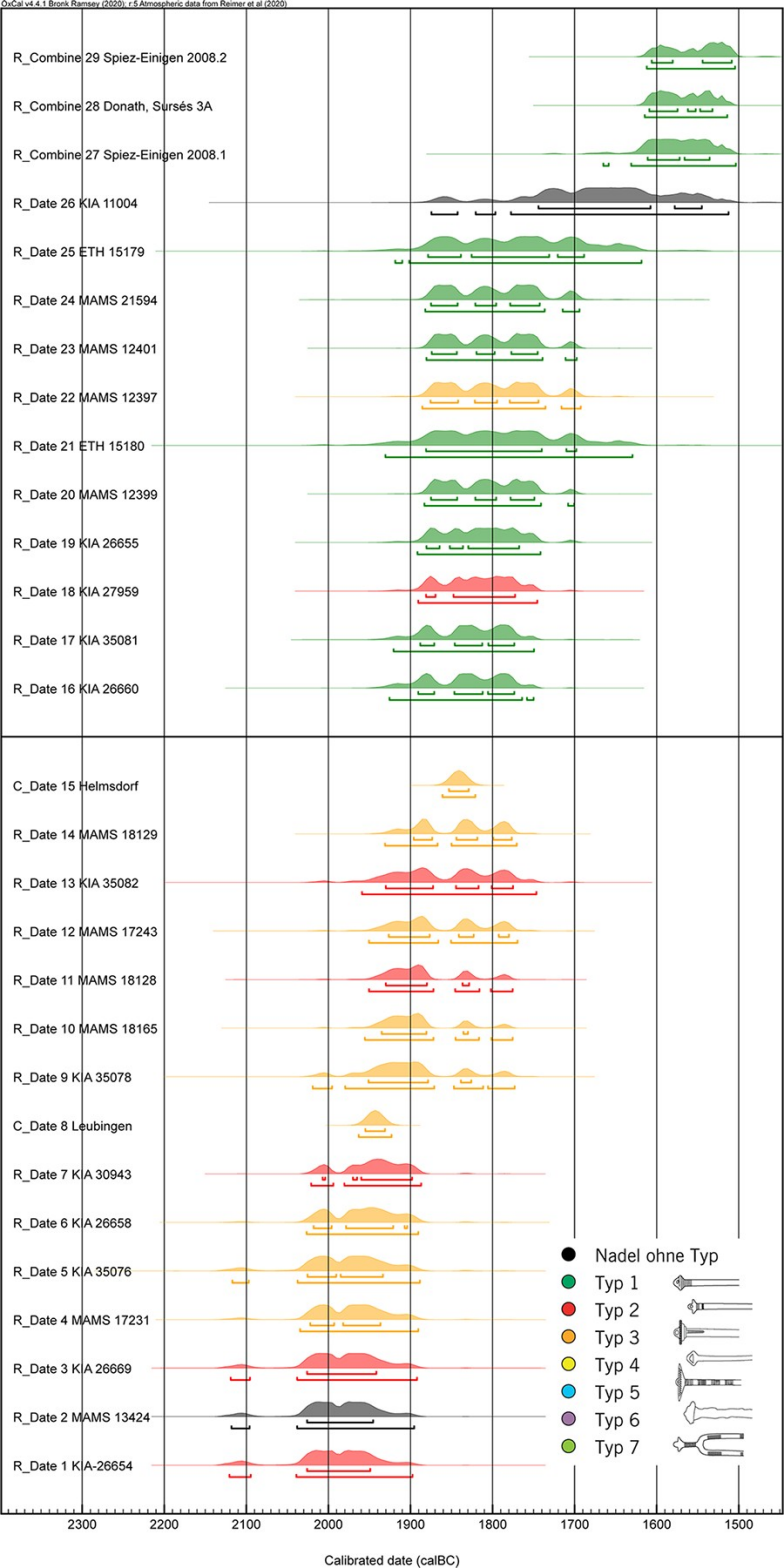
Calibrated radiocarbon and dendro dates from burials with Bronze Age eyelet pins in Europe. In Switzerland and Southern Germany only eyelet pins of type 1, 5 and 7 are found in burials. The data are from Knoll and Meller 2015 [48] and Massy 2018 [8].

The fact that in Switzerland and southern Germany the change from Bz A1 to A2 must have taken place between 1876–1820 calBC is also shown by a comparison with the Únětice area to the north east (Fig 9). Here the first Bz A2 eyelet pins date back to around 2050 calBC (Fig 10). According to Knoll and Meller 2016 these are types 2 and 3 [53]. Only in a second phase after 1900 calBC do types 1 and 7 appear. In Switzerland and southern Germany, only types 1 and 7 exist from grave contexts (Figs 9 and 10, S3 Data). On the basis of radiocarbon dating, it can be shown that only Bz A2 eyelet pins of type 2 and 3 already occur around 2050 calBc. These types do not occur in our area of investigation, but only types 1 and 7 which all occur after 1900 calBC. This observation is consistent with our model in Table 2 and Fig 8.

### Cultural changes in the third and second millennium BC

European Bronze Age archaeology traditionally divides burials according to the following categories: flat graves versus burial mounds and cremation versus inhumation. However, there is an immense variability of attendant phenomena within these burial types. These phenomena are indispensable indicators for constructing common archaeological narratives of sociocultural interaction and cultural evolution. In our study, we attempted to relate the occurrence of these phenomena to the corresponding phases of the graves. The first step was to analyze the graves purely according to their burial rites. For this analysis, we used the same dataset as before (S1 Data).

Until recently, knowledge of the EBA burial rites in the Central and Circum Alpine region was based on a small number of graves, most of which were discovered by chance and recovered unsystematically [24, 25]. Finds from graves of phase Bz A1 are known from the Swiss regions of Valais and Bernese Uplands, in particular, but most have not been analyzed due to the absence of data. With the exception of two examples that are difficult to assess–the secondary burials at Sion-Petit Chasseur, dolmen MXI and the later burial of a child in a crouched position in a stone cist, neither of which, however, is representative–no graves have been excavated to modern standards. There is therefore very little evidence as to whether people were buried in the Central and Circum Alpine region and in the Jura mountains according to Neolithic tradition in crouched positions and, depending on gender, on the left or right side, as is common in Singen and almost all of Central Europe [8]. In the Circum and Central Alpine area, this practice is documented in the above-mentioned examples from Sion, the double grave of Zurzach-Himmelreich, the cave grave of Vaduz-Hahnenspiel and possibly in grave 7 of Thun-Wiler. In the younger phase of the EBA, inhumations were in stretched positions and no sex-specific orientation could be identified [24]. Possible exceptions were the bipolar double burials of Vufflens-la-Ville VD-En Sency, Donath-Sursés, grave 3, Triesen-Fürst Johanstrasse 40 and Spiez-Einingen-Holleeweg. Capuzzo and Barceló 2015 have tried to calculate the transition from inhumation burials to cremations on the Swiss Plateau with the help of a phase model created in OxCal. They assumed that inhumations were gradually replaced by cremations and the possible transition was calculated to have taken place between 1640 and 1535 BC [49]. However, the situation became more complex from the MBA onwards, when new burial customs followed in quick succession, as a detailed article by Schmid 2019 shows [54]. His approach demonstrates that much more complex models are needed.

From 1600 BC onwards, individual graves dominated the Central Alpine region, whereas on the Swiss Plateau and in the Jura mountains, multiple burials in mounds were the most common form. Comparing Murten-Löwenberg and Fällanden, two burial patterns are noticeable. In the example of Murten, Löwenberg, there is a central grave with associated peripheral, subordinate burials. In the case of Fällanden-Fröschbach, Birmensdorf-Rameren [55] and Châbles-Les Biolleyres [56], structured communal facilities prevail, but without (recognizable) hierarchical components. In the Circum Alpine as well as in the Central Alpine area, so far as inhumations were concerned, the deceased were buried, with few exceptions, in an extended supine position. For grave 1 at Fällanden, the use of a coffin and shroud could be proven. In Lumbrein-Surin-Cresta Petschna, cremation graves were already established at the beginning of the MBA, and also occurred in the Alpine foothills from the MBA onwards [57, 58]. In Fällanden, Riehen-Britzigerstrasse Hügel 1971/2 and Weiningen-Hardwald, Hügel 3/2, cremations were placed in existing burial mounds. In southern Germany, inhumation burials are to be found in burial mounds in extended supine positions [59]. At the beginning of the LBA phase Bz D, cremations in urns dominated in the Alpine Rhine Valley as well as in the southern Alpine valleys [60]. The beginning of the LBA brought a striking change on the Swiss Plateau and in the Jura. The burials were separated from the group context of the burial mounds and "individualized". Typical for phase Bz D were cremation graves in body-length pits (Brandschüttungsgräber), which were marked with stone covers and whose sole were sometimes covered with stones. Ceramic vessels and tools are regularly found at one end of the pit, cremation and jewelry at the other, e.g. in Neftenbach-Steinmöri [59]. From the EBA to the MBA, burial rites seem to have varied from region to region all over Europe, as Schmid 2019 demonstrated [54].

**Fig 11:**
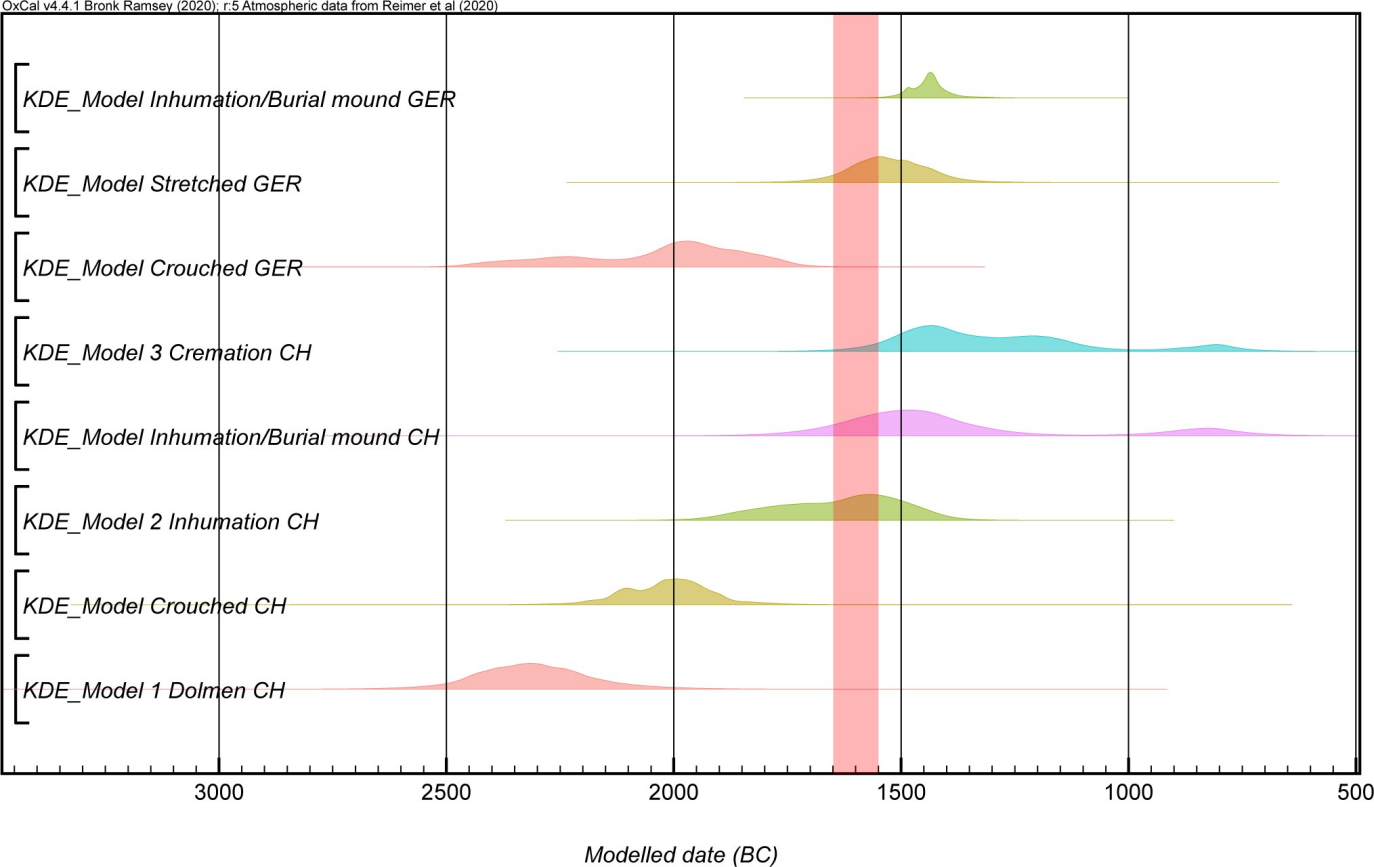
KDE_models of different regions according to their burial rites. The most striking changes take place between 1650 and 1550 at the transition from the Early Bronze Age to the Late Bronze Age. The most important of these are the emergence of burial mounds and cremations. The red bar marks the period of these changes.

As a method for summarizing radiocarbon dates for each burial category, we used kernel density models as suggested by Bronk-Ramsey 2017 [46]. The KDE_Model shows which grave customs appeared and disappeared in which regions. This model is more dynamic than the calculated phase model of Capuzzo and Barceló and can be more easily compared to Schmid’s regional distribution time series of burial types and structures [49, 54]. If we look at the results of the KDE_Models we can see significant changes from 1650 to 1550 BC (Fig 11). The most important differences are the appearance of burial mounds in the Circum-Alpine area and in southern Germany and of cremations in the Alpine area, and we can connect these with the EBA/MBA transition.

## Discussion

In general, the outcome of our chronological model for the Bronze Age agrees well with traditional Bronze Age periods. We agree with Stockhammer et al. 2015 that the beginning of the EBA is situated around 2150 BC. Stockhammer et al. 2015 assumed that pin types were regional and concluded that their evolution was not chronological. They further concluded that the form groups were principally a chorological rather than a chronological phenomenon. However, our study, which is based on a Bayesian model rather than on individual data, demonstrated that the subdivision of phases Bz A1 and A2 is purely chronological. We were able to model the phases and the transition between them, and to date the transition to between 1875 and 1820 BC (Fig 8).

**Fig 12:**
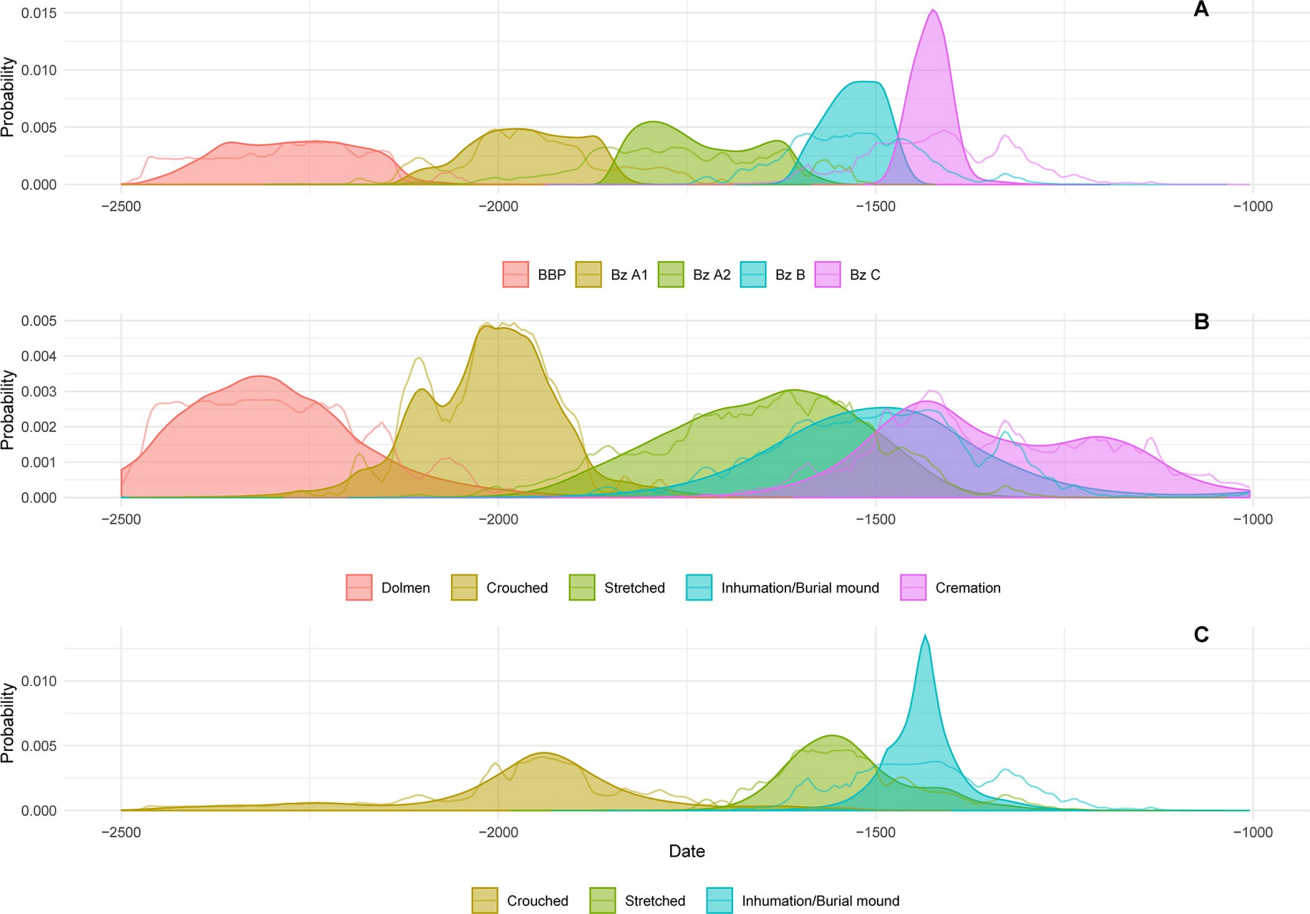
Overview of the main transformations during the European Bronze Age based on radiocarbon-dated burials. Dark colors show the kernel density plots and bright colors show the sum calibrations of the different phases (S5 File). (A) Modeled Phases in Switzerland and southern Germany; (B) KDE model of burial rites in Switzerland; (C) KDE model of burial rites in southern Germany.

The transition from EBA Bz A2 to MBA Bz B took place in the time span between 1615 and 1530 BC. Around the same time, changes in burial rites could be observed in all regions (Fig 12). The most important innovations were cremation burials and graves in burial mounds.

## Conclusions

1. The 233 radiocarbon-dated graves from southern Germany and Switzerland form an excellent starting point for a supra-regional chronological evaluation of Bronze Age graves and for assessing socio-cultural transformation processes.
2. For evaluations that go beyond the intra-site level, it is particularly important that data are collected in large numbers. Modern statistical analyses, such as sequential calibration using Bayesian methods, requiring large data collections, achieve far more sophisticated results than are possible from conventional analysis of individual data. Cultural processes must be evaluated quantitatively. Bayesian analysis is a useful tool for doing this, as it is able to determine confidence intervals and probability distributions for calibrated radiocarbon data. Information about chronology can be converted into explicit statistical estimates for dating past events.
3. Using Bayesian modelling of the above-mentioned set of 233 radiocarbon dates, our study successfully proves that the main Bronze Age phases Bz A1 and A2, Bz B and Bz C are primarily chronological.
4. In Central Europe, the LN/EBA transition and the beginning of EBA phase Bz A1 can be dated to around 2150 BC. The results of our model show a transition between EBA phases Bz A1 and A2 during the period between 1876 – 1820BC. The EBA/MBA transition set by Stockhammer et al. around 1700 BC is shown to be too early. The reason for the error was the very small number of graves (n = 3) from Bronze Age phase Bz A2 used in their study. Our model, which considered a large number of Bronze Age phase Bz A2 graves, shows that the transition took place about 100 years later, between 1615 and 1530 BC.
5. The transition to the developed BA took place after 1900 BC. The greatest change in the material culture can be seen in the different pin types. In BA phase Bz A1, there are only simple hammered pins, wire-wrapped pins and bone pins: typical pin types of this period are perforated bone pins, paddle-headed pins (Rudernadel), disc-headed pins (Scheibenkopfnadel), knot-headed pins (Schleifenkopfnadel) and Horkheim-type pins. From BA phase Bz A2 onwards, types were produced with more complex manufacturing techniques. These are eyelet pins (Ösenkopfnadeln), pins with sleeve shaped heads (Hülsenkopfnadeln), pins with cloverleaf-shaped heads (Flügelnadel) with or without decoration, pins with rhomb-shaped heads (Rautennadel) and globe-headed pins with oblique perforation (Kugelkopfnadeln).
6. In connection with the change in material culture and changes in grave offerings, a marked change in burial rituals was also noted. Individual EBA burials in flat graves were replaced by multiple MBA burials under burial mounds.

## Supporting information

S1 File Sample treatment. The description of the sample treatment at the Laboratory for the Analysis of Radiocarbon with AMS (LARA) at the University of Bern.
(TXT)

S2 File R script and data Regressive Reciprocal Averaging. Regressive Reciprocal Averaging seriation of grave inventories according to their radiocarbon dating.
(ZIP)

S3 File Code for OxCal 1. Chronological Query Language (CQL2) code for the analysis “Modelled Phases Highest posterior density intervals for the estimated start, end and KDE_plot visualization of the overall distribution of dated events within each Bronze Age phase”.
(OXCAL)

S4 File Code for OxCal 2. Chronological Query Language (CQL2) code for the analysis “KDE_models of different regions according to their rites of burial”.
(OXCAL)

S5 File R-Script oxcAAR. R-script for visualizing OxCal plots in one timeline.
(RMD)

S1 Data Supporting data and results. List of all sites and burials used in the analysis with their radiocarbon dates.
(ODS)

S2 Data Results of OxCal Code 1. Results for the analysis “Modelled Phases Highest posterior density intervals for the estimated start, end and KDE_plot visualization of the overall distribution of dated events within each Bronze Age phase”.
(CSV)

S3 Data Supporting data and results. List of all sites and burials with eyelet pins used in the analysis with their radiocarbon dates.
(CSV)

S4 Data Results of calibrated radiocarbon dates of graves with eyelet pins. (CSV)

S5 Data Results of OxCal Code 2. Results for the analysis “KDE_models of different regions according to their rites of burial”.
(CSV)
